# Rapid fabrication and dissolution of pressed ^58^Ni/Mg matrix targets for ^55^Co production

**DOI:** 10.1186/s41181-024-00324-5

**Published:** 2025-01-21

**Authors:** Jonathan Siikanen, Stefan Milton, Klas Bratteby, Wilson Lin, Jonathan W. Engle, Emma Jussing, Thuy A. Tran

**Affiliations:** 1 Department of Nuclear Medicine and Medical Physics, Karolinska University Hospital, Stockholm, 171 76 Sweden; 2 Department of Oncology and Pathology, Karolinska Institutet, Stockholm, 171 77 Sweden; 3 Department of Clinical Neurosciences, Karolinska Institutet, Stockholm, 171 77 Sweden; 4 Department of Medical Physics, University of Wisconsin, 1111 Highland Ave, Madison, WI 53705 USA; 5 Department of Radiology, University of Wisconsin, 600 Highland Ave, Madison, WI 53792 USA

**Keywords:** Cyclotron, ^55^Co ^58^Ni/Mg-matrix, Solid target, DOTA, AMA

## Abstract

**Background:**

Beyond the use of conventional short-lived PET radionuclides, there is a growing interest in tracking larger biomolecules and exploring radiotheranostic applications. One promising option for imaging medium-sized molecules and peptides is ⁵⁵Co (T₁/₂ = 17.5 h, β⁺ = 76%), which enables imaging of new and already established tracers with blood circulation of several hours. Additionally, ⁵⁵Co can be paired with the Auger-Meitner emitter ^58m^Co (T₁/₂ = 9 h, 100% IC) for radiotheranostic applications. Here we report on ^55^Co production via the ^58^Ni(p,α)^55^Co reaction channel using pressed ^58^Ni and Mg matrix targets.

**Results:**

This set up is capable to produce and isolate 240 ± 20 MBq [^55^Co]Co^+ 2^ (80% RCY) with 4 ml 0.25 M HEPES at 35 min post End Of Bombardment for 3 h, 25 µA protons irradiation. The RNP of the eluate is 99.98 ± 0.014% as measured 2 h & 17 h post EOB. AMA was determined to 1.5 ± 0.5 GBq/µmol [^55^Co]Co-DOTA at EOB. Mg dissolves rapidly in the acid mixture, leaving behind a porous, sponge-like Ni matrix increasing the surface area of the Ni and therefore accelerating the dissolution.

**Conclusion:**

We present a novel, simple, and rapid method to produce ⁵⁵Co with pressed ⁵⁸Ni/Mg matrix targets enabling faster target fabrication and dissolution. By using a simple hydraulic press, mechanically stable target coins useful for solid target irradiation are fabricated within 5 min and can be dissolved in 10 min at room temperature. The foils remain intact after irradiation and can endure irradiation conditions providing sufficient activity (> 200 MBq) for clinical doses. The method presented here using Mg as a support metal for fixation of the actual target material into target coins is applicable for other target combinations as well. Using Mg as a support metal is suitable due to its thermal conductivity, low activation, minimal impact on purification chemistry, softness, ductility, and rapid dissolution in acid.

## Background

Positron Emission Tomography (PET) has become a standard nuclear medicine modality for imaging various diseases and physiological states. PET delivers functional images that are often combined with anatomical Computed Tomography (CT) images for better positioning of the functional distribution within the body and for quantitative purposes. For clinical oncology imaging, 2-[^18^F]fluoro-2-deoxy-D-glucose ([^18^F]FDG) (Reivich et al. 1979; Ido et al. 1978), which leverages the Warburg effect (Warburg 1956; Liberti and Locasale 2016), is the golden standard and the principle and applications of [^18^F]FDG has previously been summarized (IAEA 2015, 2023, 2008). This glucose analogue is labeled with the relatively short-lived positron emitting radiohalogen ^18^F (T_1/2_= 110 min, β^+^ = 98%), a radionuclide that is suitable for labeling smaller molecules that rapidly reach and accumulate in their targets within a few hours. As an example, a typical [^18^F]FDG PET-scan is performed around 1 h post-injection (Boellaard et al. 2015).

Growing clinical interest in cell imaging (Friberger et al. 2021; Volpe et al. 2022), imaging with varied molecule sizes such as antibodies, antibody fragments (Wei et al. 2020; Dongen et al. 2021), peptides (Lewis and Anderson 2007) and the concept of radiotheranostic (Bodei et al. 2022) i.e., the pairing of diagnostic and therapeutic molecules for precision medicine (Arnold 2022) has driven the demand for longer-lived and chemically distinct radiometals, such as ^89^Zr (T_1/2_= 78 h, β^+^ = 24%). This also includes radiometals with shorter half-lives like ^68^Ga (T_1/2_= 68 min, β^+^ = 87%) (Jussing et al. 2021; Siikanen et al. 2021). Radiometals simplifies the linking to the targeting vectors via chelation under the necessary mild conditions required for most biomolecule-vectors.

One radiometal useful for imaging medium-sized molecules and peptides, with blood circulation of several hours is ^55^Co (T_1/2_=17.5 h, β^+^ = 76%) (Mitran et al. 2017). This radiometal offers the possibility for imaging of new upcoming tracers (Mitran et al. 2017) but it is also useful as an alternative to some of the already established tracers where the image contrast could benefit from longer circulation times. Also, ^55^Co has the potential to be used for radiotheranostic purposes when pairing with the Auger-Meitner electron emitter ^58m^Co (T_1/2_= 9 h, 100% IC) (Thisgaard et al. 2011; Barrett et al. 2021). Furthermore, the longer half-life of ^55^Co facilitates logistics when shipping to distant labs.

For PET-cyclotrons, ^55^Co can be accessed via the ^58^Ni(p,α)^55^Co and ^54^Fe(d, n)^55^Co routes (Barrett et al. 2021). When preparing cyclotron targets with ^58^Ni or ^54^Fe, the golden standard is to electroplate the material to a metallic supportive backing, a process that requires many hours to complete. The plated coin is then positioned in the beam of charged particles and cooled with water on the back side. For ^55^Co production via irradiation of Ni it is also feasible to use natural Ni because the relatively high abundance of ^58^Ni (58%) which is not the case for the ^54^Fe (6%) set up that requires enriched material. The production of ^55^Co using ^nat^Ni or enriched ^58^Ni has previously been reported (Barrett et al. 2021; Mastren et al. 2015; Valdovinos et al. 2017) where the purification of [^55^Co]Co^+ 2^ from Ni required dissolution times of 0.3–1 h for electroplated Ni targets and about 4 h for Ni foils using strong hydrochloric acid (HCl) and additional heating. The electroplated targets benefit from the heterogenous surface with a greater surface area exposed to the HCl than the intact foils, therefore speeding up the dissolution process.

This work presents a novel target method for producing ^55^Co via the ^58^Ni(p,α)^55^Co-reaction. Here, nickel powder is mixed with magnesium powder and pressed together using a simple hydraulic press to form target coins useful for solid target irradiation. Due to the softness and ductility of the magnesium a mixed matrix of the two metals can easily be formed. Irradiation of Mg with ~ 13 MeV protons only yields short lived ^25^Al(T_1/2_=7 s) and some small amounts of ^22^Na (T_1/2_=2.6 y) (IAEA 2019). Also, Mg is easy to dissolve and has a low distribution coefficient for a conventional AG1-X8 anion resin. Furthermore, Mg is a better heat conductor than Ni and should assist in the heat transfer within the target coin.

The aims of using these pressed targets are to; facilitate the manufacturing of mechanically stable foils, eliminate the need of heating and to speed up the dissolution process. We anticipate a faster dissolution rate due to the presence of the Mg component in the mixed foils which will dissolve rapidly in the acid mixture, leaving behind a porous, sponge-like Ni matrix with an increased surface area to the remainder of the acid solution. Furthermore, focus has been to automate the purification process and to assess the Apparent Molar Activity (AMA) on the eluate using DOTA (1,4,7,10-Tetraazacyclododecane-1,4,7,10-acetic acid) titrations.

## Methods

### Targetry and cyclotron irradiation

Enriched nickel, ^58^Ni, was supplied as metal powder with various grain sizes (CortecNet, France). Isotope enrichment and other metal impurities as provided from the supplier are shown in Table 1.

**Table 1:** Characteristics of the enriched ^58^Ni

Isotope	^58^Ni	^60^Ni	^61^Ni	^62^Ni	^64^Ni
Enrichment (%)	99.905	0.083	0.004	0.004	0.004
Chemical admixture					
**Element**	**Al**	**Co**	**Cr**	**Cu**	**Fe**
Content (ppm)	< 10	< 10	< 10	< 10	< 10
**Element**	**Mg**	**Mn**	**Pb**	**Si**	**Ti**
Content (ppm)	< 10	10	< 10	< 10	< 10
**Element**	**Zn**	**C**	**S**	**P**	
Content (ppm)	< 10	104	< 10	2.4	

Before irradiation experiments, various test foils were made by mixing different combinations of Mg/^nat^Ni mass ratios (0.50/0.50, 0.40/0.60, 0.25/0.75, 0.10/0.90 and 0.00/1.00) to find a good balance between mechanical stability by adding Mg while preserving production capacity i.e. keeping the Ni fraction high. The various mixtures were poured into a 15 mm inner diameter hardened steel pressing die set (Across International). To even out the powder, the die set was gently shaken before using the push rod to twist and further smooth the powder mixture. The die set was then pressed with 20 ton (1.1 GPa) with a simple manually operated hydraulic press. The mechanical stability of the foils was tested by finger force. The final combination with satisfying mechanical stability contained 150 mg of ^nat^Ni (powder < 50 μm, 99.7%, #266981, Sigma Aldrich) or enriched ^58^Ni and 50 mg of Mg (powder ≥ 99%, #13112, Sigma Aldrich) resulting in approximately 0.3 mm thick foils. This Mg/Ni ratio was used for all irradiation and chemistry experiments.

Two different solid target platforms mounted to two different General Electric’s PETtrace 800-series cyclotrons in Madison and Karolinska were used to test the thermo-mechanical performance of ^nat^Ni/Mg-foils (Madison and Karolinska). Later enriched ^58^Ni/Mg foils were used to collect data (Karolinska).

### Karolinska lab

**Fig. 1:**
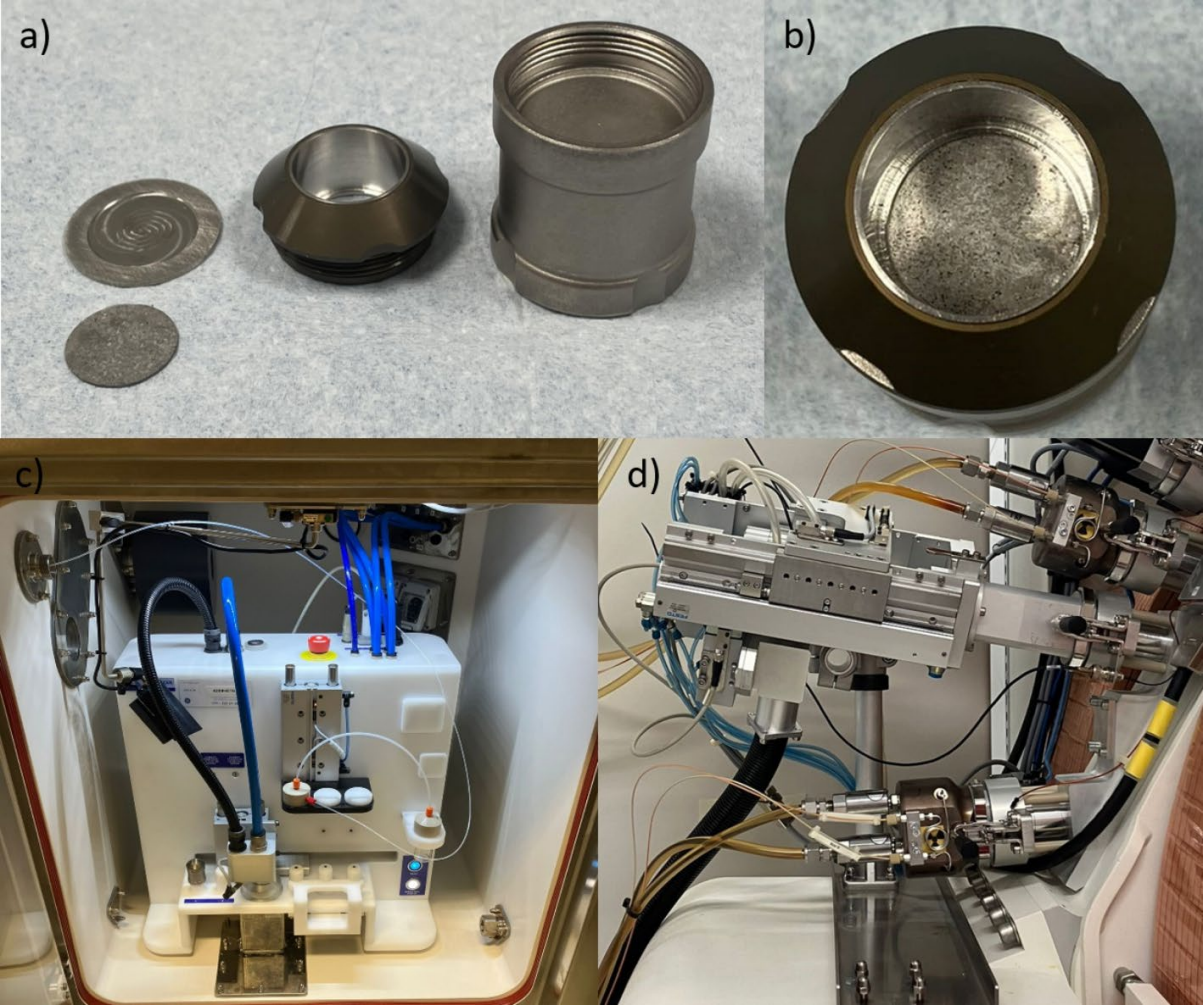
The production set up at Karolinska lab including **a**) and **b**) enriched ^58^Ni foil together with dismounted and mounted shuttle with Pt-backing, Pt-coated hat and shuttle body, **c**) shuttle transfer system (Comecer EDS) and **d**) solid target (Comecer PTS)

Foils were placed in a shuttle and pneumatically transferred from a hotcell, using a transfer module (Comecer EDS), to the cyclotron’s irradiation station (Comecer PTS) (Fig. 1) which applies He-cooling to the front of the pressed target and water cooling to the back wall of the shuttle as described before (Siikanen et al. 2021). To investigate the overall performance, foils were irradiated with 10, 18 or 25 µA, 12.6 MeV protons for 1–180 min including visual inspection, after appropriate decay, in between runs. This solid target is beam limited to 25 µA protons, which was the final current used to irradiate the foils for up to 180 min before being retrieved to the same hotcell. The operations were automated and remotely performed, which decreased radiation exposure to the personnel.

### Madison lab

**Fig. 2:**
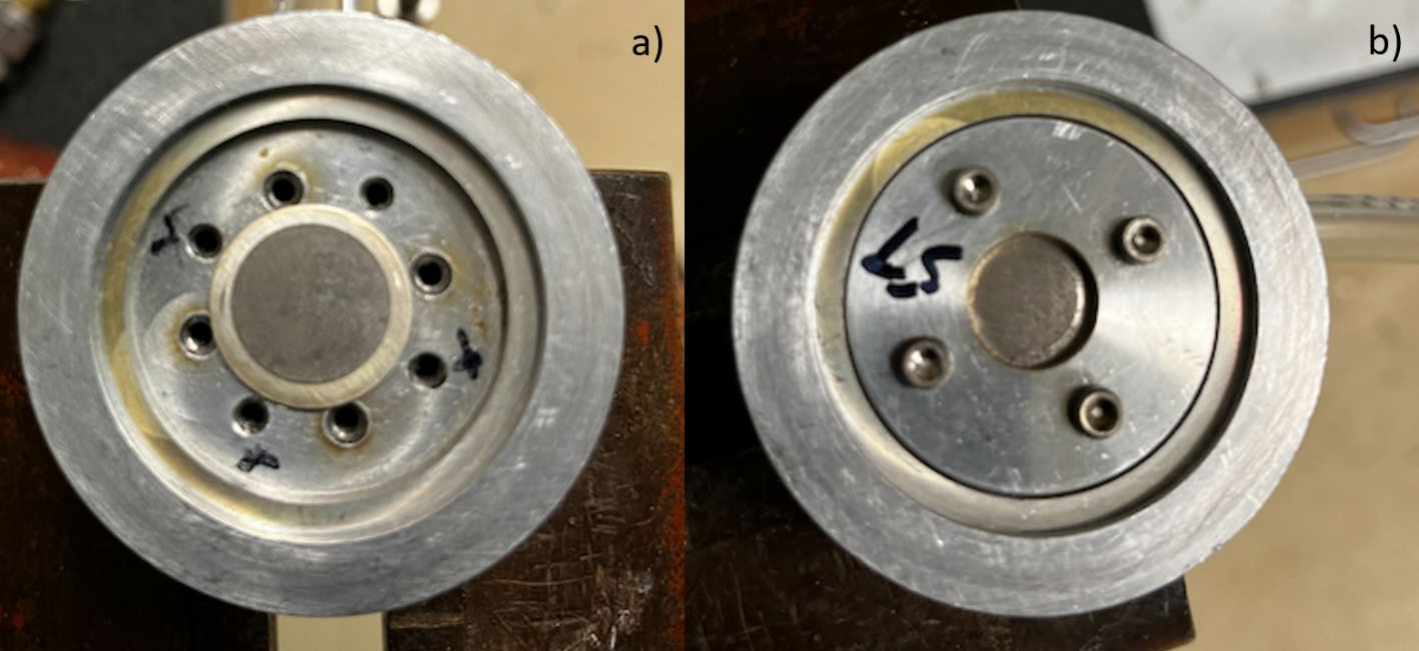
The home built solid target holder in Madison where the target coin (**a**) is placed and (**b**) pressed against a water-cooled Ag-backing with a clamp down washer

To further test the performance of the foils, a home-built solid target system was used allowing for higher beam currents than 25 µA. Here, a clamp down washer pressed foils to an Ag backing disc placed in a home-built capsule/solid target system (Fig. 2a & b) for foil irradiations using only water cooling. The foils were exposed to 25–30 µA, 16 MeV beam for varying lengths of time with rear cooling from a water jet and without helium cooling on the front of the Ni/Mg disc.

### Purification of [^55^Co]Co^+ 2^

**Fig. 3:**
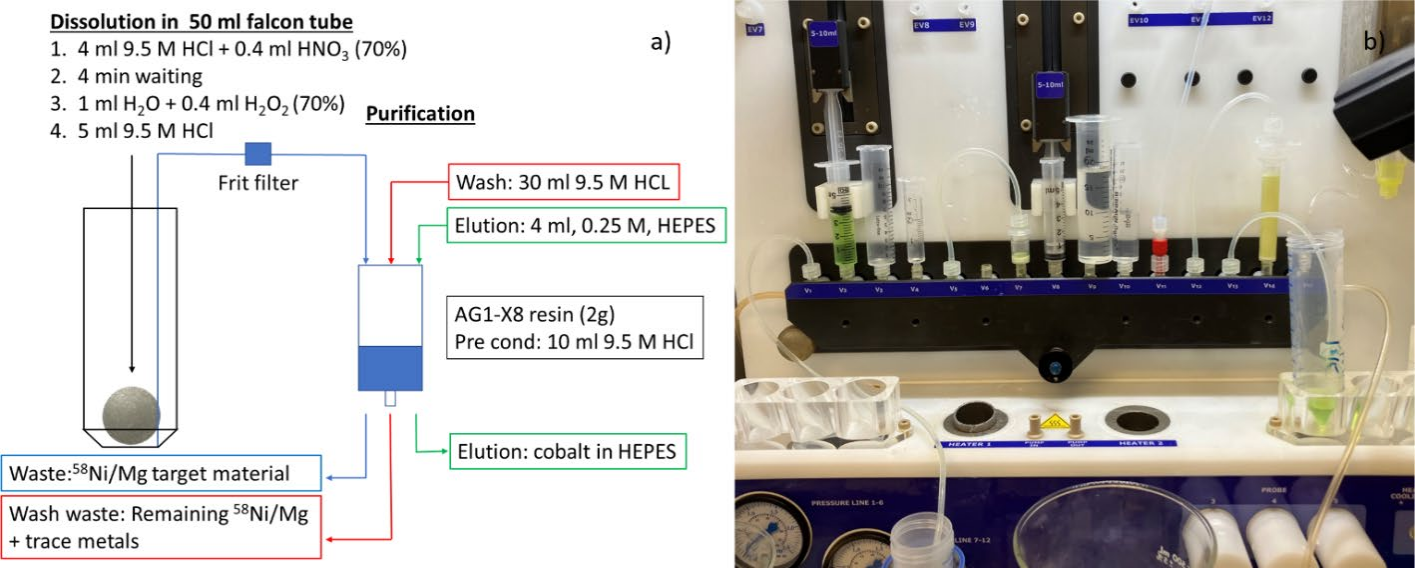
**a**) Schematic showing the dissolution and purification process of [^55^Co]Co^+ 2^ from Ni/Mg targets which was automated **b**) using a separation module (Comecer, Taddeo, PRF)

Purification experiments were conducted at Karolinska where the separation of [^55^Co]Co^+ 2^ from Nickel and Magnesium was programmed and automated with a separation module (Comecer, Taddeo PRF, Fig. 3b). Irradiated foils were placed in a 50 ml Falcon tube and dissolved by the addition of HCl (4 ml, 9.5 M) mixed with HNO_3_ (0.4 ml, 70% HNO_3_). After 4 min, a mixture of 1 ml H_2_O (TraceSelect) and 400 µl H_2_O_2_ was added. After 10 min total dissolution time, HCl (5 ml, 9.5 M) was added to the solution and then the entire volume was passed over a frit-filter and then over an AG1-X8 anion exchange resin (2 g); previously pre-conditioned with 10 ml of 9.5 M HCl. After trapping of [^55^Co]Co^+ 2^, the resin was washed with 10 ml of 9.5 M HCl and dried for 4 min with N_2_ before eluting [^55^Co]Co^+ 2^ with 4 ml of 0.25 M HEPES (see Fig. 3a). Separation yield was determined as activity in the eluate divided by activity in eluate, resin, and wash solution at the End of Bombardment (EOB). The eluate to foil activity was also determined (EOB). The amount of Ni^2+^ that followed [^55^Co]Co^+ 2^ into the eluate was assessed with semi-quantitative test strips (Ni^+ 2^ test strips, Quantofix). Activity and half-life were determined with a Capintec CRC15 dose calibrator using factory calibration number 481.

### Radio nuclidic purity (RNP)

Gamma spectrometry of a dissolved target fraction was performed before purification 1 h post EOB and of a purified ^55^Co fraction at 2 and 17 h post EOB using a high-purity germanium detector (Canberra with Cryo-Cycle II Hybrid Cryostat) (with dead times < 5%). The detector had previously been energy and efficiency calibrated (0.5 m distance) with a calibration source (5 ml flame-sealed vial, catalog no. 7600, Eckert and Ziegler) containing 17 gamma lines (88-1836 KeV). The fractions were diluted to 5 ml and placed in equivalent vials as the calibration source to minimize geometry-related variation in quantification, between calibration source and fraction. Gamma-ray intensities and energies were picked from nuclear decay data in the MIRD format (NNDC 2023). Data was processed using Genie-2000 spectroscopy software (Mirion Technologies). To quantify the amount of co-produced ^22^Na the entire waste from a purification was analyzed two weeks after irradiation.

### Metal analysis and titrations with DOTA

Apparent Molar Activity (AMA) of the eluate containing [^55^Co]Co^+ 2^ was assessed via titration using DOTA chelator (1,4,7,10-Tetraazacyclododecane-1,4,7,10-acetic acid, Sigma-Aldrich). Solutions of DOTA were prepared by serial dilution (range 0.244 nmol – 0.125 µmol). DOTA-titrations (*n* = 3) were performed in 0.1 M HEPES adjusted to pH 7.0 using 2 M NaOH in Eppendorf tubes containing a total volume of 500 µL. The solutions were made by a serial dilution of 1:1 for each concentration with DOTA dissolved in 0.25 M HEPES buffer (200 µL). To each reaction 300 µL of the diluted ^55^Co-eluate was added, the activity was measured, and the reactions were incubated at 90 °C for 60 min in a thermoshaker (Eppendorf Thermomixer C) at 550 rpm. At the end of the reaction, a sample was taken for analysis by iTLC to quantify the complexation of [^55^Co]Co-DOTA. The iTLC stationary phase was 10 × 2 cm silica gel RP plates (TLC Silica gel 60 RP-18 F_254s_, Merck) eluted with 0.5 M triethylammonium acetate (Et3NHAc):50% methanol in water 1:1, pH 5.5, were the [^55^Co]Co-DOTA has a Rf-value of > 0.35 and the free unchelated [^55^Co]Co^+ 2^ has an Rf-value of < 0.10. Analysis of the iTLC plates was carried out using a TLC scanner (AR-2000 TLC Scanner, with software WinScan 3.0, Eckert & Ziegler). The percentages of RCP were plotted as a function of DOTA (µmol). AMA was determined based on complexation (i.e. RCP) at 50% and then by dividing these values by 2, as suggested earlier (IAEA-TECDOC-1863 2019). The values were decay corrected to End of Bombardment (EOB).

## Results

**Fig. 4:**
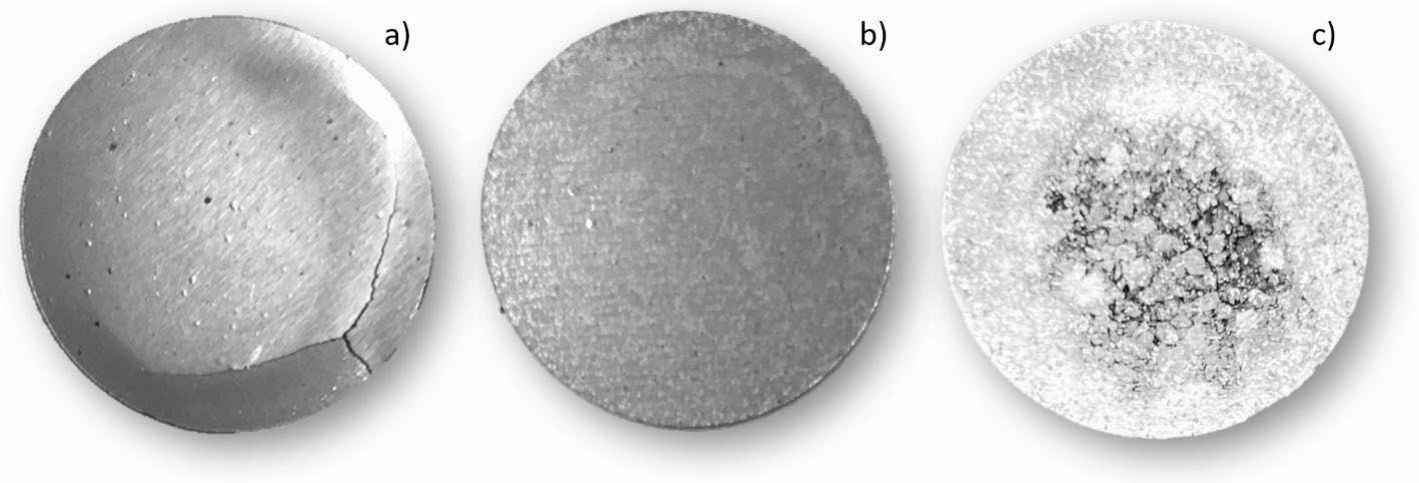
(**a**) a ^nat^Ni (200 mg) foil (**b**) pressed ^nat^Ni (150 mg)-Mg (50 mg) foil and (**c**) a ^58^Ni (150 mg)-Mg (50 mg) foil. The foils are 0.3 mm thick and have a diameter of 15 mm

Examples of pressed Ni and Ni/Mg foils are shown in Fig. 4. A summary of results is presented in Table 2.

**Table 2:** Results (Karolinska lab) from irradiations (3 h, 25 µA, 12.6 MeV p) on 150/50 mg ^58^Ni/Mg foils

Category	Results	Comment
Produced A in foil	300 ± 10 MBq	A_sat = 120 MBq/µA
Total dissolution time	10 min	Includes HCl/NH_4_ and H₂O/H₂O₂
Isolated [^55^Co]Co^+ 2^	240 ± 20 MBq	35 min post EOB*
RCY	80 ± 2%	Yield = isolated / (waste + resins + isolated)
Isolated ^55^Co/Foil ^55^Co	> 70%	Loss mainly due to un-dissolved larger grains
Ni in eluate	0 mg/L	Read out on semi-quantitative strips
AMA (DOTA)	1.5 ± 0.5 GBq/µmol	(3.0 ± 0.9 GBq/µmol x 50%)
RNP eluate	99.98 ± 0.014%	Measured 2 h & 17 h post EOB
T_1/2_	17.48 h	17.53 h (Nudat3)
pH	≤ 1	Read out by pH strips

*5 min shuttle drying, 10 min dissolution and 20 min purification. All values are decay corrected to EOB

**Fig. 5:**
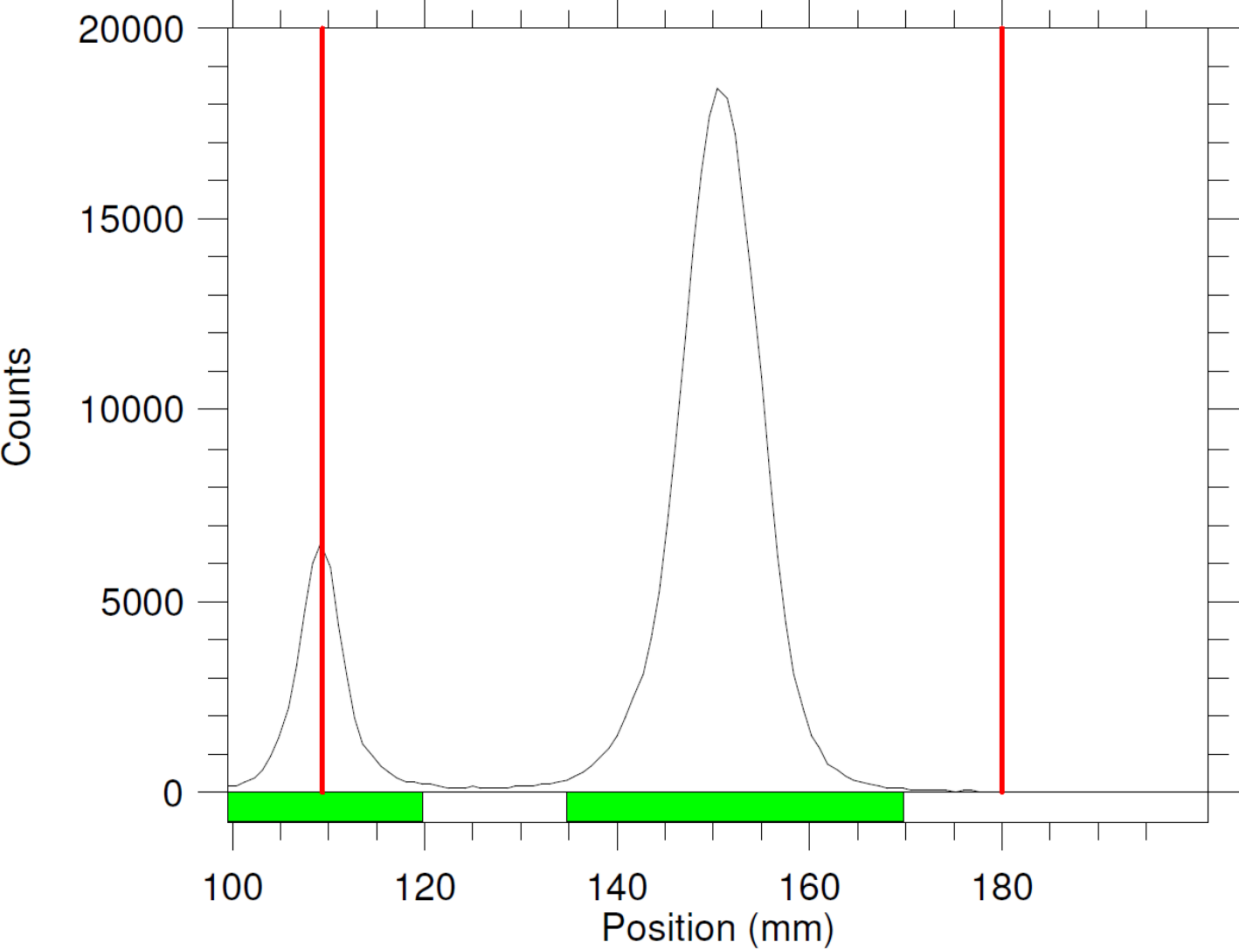
Example of typical TLC chromatogram data point (Titration #1, DOTA mass 0.00391 µmol). Red lines represent application point and solvent front

A summary of results from titrations is shown in Table 3. An example of TLC chromatogram generated during the titration is shown in Fig. 5.

**Table 3:** Summary of results from titrations with DOTA

DOTA (µmol)	Titration #1 RCP (%)	Titration #2 RCP (%)	Titration #3 RCP (%)
0.03125	97.6	99.1	97.3
0.01563	97.3	99.7	92.2
0.00781	95.9	92.2	78.9
0.00391	82.2	85.8	39.7
0.00195	39.2	22.9	16.5
0.00098	19.9	8.0	5.5

## Discussion

### Foil manufacturing

As shown in Fig. 4a) the 100% Ni foils were fragile containing cracks and were not suitable for bombardment because of the risk of foil fragmentation inside the solid target system. Homogenous, natural Ni powder mixed with Mg (Fig. 4b) resulted in more mechanically stable and better mixed foils as compared to when enriched nickel, with various grain sizes, was mixed and pressed (Fig. 4c). To better smooth out various grain sizes it should be possible to grind down larger particles using a Cryogenic Mill or similar. However, this was not applied in this project. Since the heat conductivity of Mg (160 W·m^-1^K^-1^) is almost three times higher than for Ni (60 W·m^-1^K^-1^) the heat transfer within the target coin should be improved compared to a Ni-only pressed foil.

### Target testing and production of ^55^Co

At the Karolinska lab, foils were placed on the platinum-backing and placed in the shuttle body (Fig. 1a & b). The platinum coated hat was screwed on top to press down the foil and seal the contact with an O-ring. This created a chemically inert cavity useful for dissolution with strong acids directly after receiving the shuttle back from the irradiation position. However, this possibility was not included in these initial tries since the dissolution media used here also dissolved platinum. Instead, the foils were dismounted manually and transferred with pliers to the automated separation process. Repeated successful mounting and dismounting showed that the foils were mechanically stable and suitable for this solid target set up. However, it was possible to break the foils using finger force, so some cautious handling was necessary. Foils were completely intact without any burn marks after the beam intensity escalation study and the longer irradiations of up to three hours.

At the Madison lab only ^nat^Ni/Mg mixed foils were tested. One of the foils was exposed to water overnight. The disc pitted and seemed to corrode slightly during this time, so it was not considered possible to clamp it to a target without some additional backing. Next, a foil was pressed into a variety of backing materials (Nb, Ag, Au) with a hydraulic press, without success forming a mechanically adherent bond. A clamp down washer was therefore used to press foils to an Ag backing disc placed in a home-built capsule for foil irradiations (Fig. 2). As the beam current was increased to 30 µA, a small area of the surface of the inter metallic changed confirmation to a shiny area which indicated that the target just had begun to melt. This was further verified by a coincident drop in the cyclotron’s vacuum pressure, and a small perturbation in the measured and logged neutron flux in the vault. However, when the target was removed from the cyclotron the foils could easily be separated from the backing and maintained its structure. With He-cooling, we anticipate that tolerated beam intensities would easily reach 35 or 40 µA.

It has previously been reported that approximately 40 MBq of ^55^CoCl_2_ was administered to humans for pharmacokinetic and dosimetry studies of ^55^Co and ^57^Co (Jansen et al. 1996). In this work, the production capacity of 200–300 MBq of ^55^Co should provide enough activity for a single or a few patient doses. The exact number of doses depends on factors such as the specificity of the molecule used, the PET scanner’s sensitivity, and the scan duration. High-specificity tracers and sensitive scanners enable lower doses per patient, thus increasing the number of potential scans.

### Radio nuclidic purity

Because of the high ^58^Ni-enrichment with only small amounts of ^64^Ni and ^61^Ni (0.004%), ^64^Cu (T_1/2_=12.7 h) and ^61^Cu (T_1/2_=3.3 h), was not observed in the gamma spectrum as seen when using ^nat^Ni. Some small amount of ^60^Cu (T_1/2_=23 min) was found in the non-purified sample due to the larger ^60^Ni fraction (0.083%) in the enriched ^58^Ni material. This ^60^Cu had decayed away when measuring the purified sample. For a single AG1-X8 resin, Cu can be separated from Co using different HCl concentrations as eluant as described before (Avila-Rodriguez et al. 2007) but this was not implemented in this setup. The major drawback of using the ^58^Ni(p,α)^55^Co reaction is the surprisingly high cross section of ^58^Ni(p,2p)^57^Co reaction (Reimer and Qaim 1998) which might cause radiation protection issues due to the long lived ^57^Co (T_1/2_=270 d). Due to ^57^Co contamination (approx. 50 kBq) of the eluates it was not possible to ship them out for inductively coupled plasma-mass spectrometry analysis.

The proton irradiation of Mg produces ^25^Al(T_1/2_=7 s) and some small co-production (approx. 1 kBq/µAh for this setup) of ^22^Na (T_1/2_=2.6 y) via the ^25^Mg(p,α)^22^Na reaction. Even though ^22^Na does not contaminate the purified eluate for the given separation process it should be handled according to local rules regarding radioactive waste management.

### Separation of [^55^Co]Co^+ 2^ and apparent molar activity

For our dissolution procedure (HCl, HNO_3_, H_2_O_2_), the Mg component in the mixed foils dissolved rapidly in the acid mixture, leaving behind a porous, sponge-like Ni matrix. This increased the surface area of the Ni matrix, accelerating the dissolution. This process was much slower for the foil pressed from pure Ni powder (not fully dissolved after > 3 h). The reason for the low eluate to foil activity ratio is explained by the larger grains of enriched ^58^Ni, which could not be homogeneously mixed with the Mg and therefore could not be dissolved rapidly. This concern was not observed when testing with ^nat^Ni since it consisted of a homogeneously finer powder. Semi quantitative strips, with limited detection limit, indicated no contamination of Ni^+ 2^ (0 mg/ml) in the eluate and DOTA titration showed quantitative conversion for higher concentrations of DOTA. AMA result of 1.5 ± 0.5 GBq/µmol [^55^Co]Co-DOTA (EOB) is in agreement to previously reported values for a single AG1-X8 purification set-up (Mastren et al. 2015) which indicates negligible influence of Mg^+ 2^.

## Conclusion

We present a novel easy and efficient method to produce ⁵⁵Co via ⁵⁸Ni, using pressed ⁵⁸Ni/Mg mixed matrix targets for easy and rapid target manufacturing and dissolution. With this approach mechanically stable target coins suitable for solid target irradiation are fabricated within 5 min using a simple hydraulic press and the foils can be dissolved in 10 min at room temperature. The foils retained their structure and were intact after handling and irradiation. Without any optimized thermal coupling these foils can at least withstand beam currents of 25 µA for 3 h, as verified from two different labs/solid targets giving enough activity (> 200 MBq) for a single or a few patient doses. The method presented here using Mg as a support metal for fixation of the actual target material into target coins is applicable for many other target combinations. Using Mg as a support metal is suitable due to its thermal conductivity, low activation, minimal impact on purification chemistry, softness, ductility, and rapid dissolution in acid.

## Data Availability

Presented in the main paper.

## Abbreviations

PET: Positron Emission Tomography
CT: Computed Tomography
[^18^F]FDG: 2-[^18^F]fluoro-2-deoxy-D-glucose
HCl: Hydrochloric Acid
AMA: Apparent Molar Activity
DOTA: 1,4,7,10-Tetraazacyclododecane-1,4,7,10-acetic acid
MeV: Mega electron Volt
µA: Micro Amperes
HNO_3_: Nitric Acid
H_2_O_2_: Hydrogen Peroxide
HEPES: 4-(2-hydroxyethyl)-1-piperazineethanesulfonic acid
EOB: End Of Bombardment
RNP: Radio Nuclidic Purity
NAOH: Sodium Hydroxide
iTLC: Instant Thin Layer Chromatography
Et3NHAc: Triethylammonium acetate
RF: Retention Factor
RCP: Radiochemical Purity
RCY: Radiochemical Yield
MBq: Mega Becquerel
pH: Potential of Hydrogen
